# An Explosive Epidemic of DENV-3 in Cairns, Australia

**DOI:** 10.1371/journal.pone.0068137

**Published:** 2013-07-16

**Authors:** Scott A. Ritchie, Alyssa T. Pyke, Sonja Hall-Mendelin, Andrew Day, Christopher N. Mores, Rebecca C. Christofferson, Duane J. Gubler, Shannon N. Bennett, Andrew F. van den Hurk

**Affiliations:** 1 School of Public Health, Tropical Medicine and Rehabilitative Sciences, James Cook University, Cairns, Queensland, Australia; 2 Tropical Regional Services, Cairns Population Health Unit, Queensland Health, Cairns, Queensland, Australia; 3 Public Health Virology, Queensland Health Forensic and Scientific Services, Coopers Plains, Queensland, Australia; 4 School of Veterinary Medicine, Department of Pathobiological Sciences, Louisiana State University, Baton Rouge, Louisiana, United States of America; 5 Program on Emerging Infectious Diseases, Duke-National University of Singapore Graduate Medical School, Singapore; 6 Department of Tropical Medicine, Medical Microbiology and Pharmacology, John A. Burns School of Medicine, University of Hawaii at Manoa, Honolulu, Hawaii, United States of America; 7 Department of Microbiology, California Academy of Sciences, San Francisco, California, United States of America; University of Texas Medical Branch, United States of America

## Abstract

From November 2008-May 2009 Cairns Queensland Australia was struck by an explosive epidemic of DENV-3 that exceeded the capacity of highly skilled dengue control team to control it. We describe the environmental, virological and entomological factors associated with this outbreak to better understand the circumstances leading to its occurrence. Patient interviews, serological results and viral sequencing strongly suggest that the imported index case was infected in Kalimantan, Indonesia. A delay in notification of 27 days from importation of the index case until Queensland Health was notified of dengue transmission allowed the virus to amplify and spread unchecked through November 2008. Unseasonably warm weather, with daily mean temperatures exceeding 30°C, occurred in late November and would have shortened the extrinsic incubation period of the virus and enhanced transmission. Analysis of case movements early in the outbreak indicated that the total incubation period was as low as 9–11 days. This was supported by laboratory vector competence studies that found transmission by *Aedes aegypti* occurred within 5 days post exposure at 28°C. Effective vector competence rates calculated from these transmission studies indicate that early transmission contributed to the explosive dengue transmission observed in this outbreak. Collections from BG sentinel traps and double sticky ovitraps showed that large populations of the vector *Ae. aegypti* occurred in the transmission areas from November – December 2008. Finally, the seasonal movement of people around the Christmas holiday season enhanced the spread of DENV-3. These results suggest that a strain of DENV-3 with an unusually rapid transmission cycle was able to outpace vector control efforts, especially those reliant upon delayed action control such as lethal ovitraps.

## Introduction

Dengue is the leading arboviral cause of morbidity worldwide, with an estimated 390 million infections occurring annually [1]. There is currently no vaccine, and dengue control is limited to vector control and community engagement/public education programs. The primary vector of urban dengue, the mosquito *Aedes aegypti,* utilizes artificial containers for immature development while adults prefer to harbor within man-made premises. The control of *Ae. aegypti* typically involves ‘house-to-house’ treatment of water-holding containers and interior residual spraying (IRS) of premises [2], [3]. Source reduction campaigns consisting of removal of potential water holding containers are also employed [4].

Large dengue epidemics are particularly costly. A delay in recognition of a dengue epidemic can exponentially increases the total number of cases and total cost to the community [5]. Despite a relatively low mortality rate, the large number of cases cumulatively creates relatively high disability-adjusted life year (DALYs) values for dengue. A mean loss of 658, 465 and 127 DALYs per million individuals annually has been reported in Puerto Rico [6], Thailand [7] and Latin American+Caribbean regions [8], respectively.

Dengue has emerged as a leading arboviral health issue in Australia, with hundreds of imported cases annually, and local transmission resulting in multiple outbreaks in northeastern Queensland [9], [10]. A large multi-city outbreak of DENV-2 in 1992–93 led to the development of the Dengue Fever Management Plan (DFMP) by Queensland Health (QH; http://www.health.qld.gov.au/dengue/managing_outbreaks/default.asp) in 1994. Since this time (1995–2012) there have been 42 outbreaks comprised of 3,086 confirmed dengue cases and three deaths; the majority (37 outbreaks and 2,364 cases) have occurred since 2000. Queensland Health’s DFMP has generally been successful in constraining outbreaks, eliminating dengue viruses and preventing dengue from becoming endemic. Only 5 (14%) of the dengue outbreaks have exceeded 100 confirmed cases, and 19 (53%) of the outbreaks were restricted to less than 8 weeks duration. Most of the cases (2529, 82%) have come from sporadic large outbreaks (n = 5). The vector control program within the DFMP focuses on insecticidal treatment of containers and IRS in response to active dengue cases [2]. A public education program emphasizes the need for residents to maintain clean yards and eliminate receptacles that could serve as larval habitats. A limited source reduction campaign undertaken by vector control staff supplements these activities. The causes of sudden explosive transmission leading to widespread epidemic dengue are manifold [11]. Reduced herd immunity, caused in part by a successful decade-long dengue control program, has led to the resurgence of dengue epidemics in Singapore [12]. On a shorter time scale, high temperatures that reduce the extrinsic incubation period (EIP) in the vector *Ae. aegypti* can lead to sudden increases in transmission rate [13], [14]. Heavy rainfall and associated high humidity and vapor pressure can increase both vector production [15] and survival [11], increasing the risk of dengue transmission. Conversely, dry weather can amplify dengue transmission by increasing water hoarding and production of *Ae. aegypti*, especially in close association with humans, increasing vector densities that potentiate dengue outbreaks as observed in Brazil [16] and in Barbados [17]. Changes in the viral genome due to mutation and selection can produce dengue strains that have greater epidemic potential and virulence [18]–[21] and that replicate faster within the mosquito, as was the case with chikungunya virus (CHIKV) [22], [23], and DENVs [24]–[25].

In 2008 an explosive epidemic of DENV-3 resulted in the first publically declared dengue epidemic in Australia since the DFMP was launched in 1994. The outbreak caused 931 confirmed cases and one death, and cost Queensland Health ca. $AUS3 mil. in direct costs [5]. The epidemic spread was especially rapid, with a basic reproduction number (Ro; the number of secondary cases generated per case) of 2.19, and an effective reproductive number (R*t*; relative increase in cases over a 14 day incubation period) ranging from 2–12 during the first 2 months of the epidemic [5]. This particular dengue outbreak overwhelmed vector control staff, with an additional 50–60 field personnel required to conduct vector control.

What was unique about this dengue ecosystem that resulted in an outbreak that overwhelmed a well-funded, experienced and historically successful dengue control program? In response to this overarching question, we: a) investigated epidemiological, environmental and entomological features of the outbreak, b) undertook genetic analysis of the virus strain responsible, and c) studied the vector competence of the Cairns *Ae. aegypti* for the 2008 epidemic strain and a strain of DENV-3 which circulated in the Cairns region from 1997 to 1998 [10]. This later outbreak was also severe, resulting in 498 confirmed cases, 20% of which were hospitalized, in three communities over a 70 week period. These investigations provide valuable insight into what caused the magnitude of the epidemic and how it could potentially influence dengue control strategies in the future.

## Materials and Methods

### Dengue Case Definition and Epidemiology

Queensland Health was notified of suspected dengue cases (i.e., detection of dengue IgM antibodies or viral RNA in serum samples) by medical practitioners and pathology laboratories. The test results, name, address, phone numbers and specific symptoms for each case were then supplied to QH public health nurses, who immediately conducted contact tracing telephonic interviews to determine a patient’s travel history and onset of illness (used to estimate date of infection). Furthermore, they obtained the most likely place of infection by epidemiologically-linking the places visited (home, work or other places) during the 4–7 days before the onset of illness [27]. Locations where patients recalled mosquito biting and addresses with confirmed dengue the previous 2–4 weeks were considered a likely place of infection. For two or more epidemiologically-linked locations, the most likely acquisition site was based on the relative amount of daylight time spent at the address. The most likely place of infection was updated if additional information was obtained (i.e., confirmed dengue cases in an area). Dengue cases and case data were compiled on a database and analyzed in Excel.

### Entomological and Environmental Conditions Associated with the Outbreak

*Aedes aegypti*, the only competent dengue vector in the Cairns region, was sampled using Biogents Sentinel (BGS) traps [28], [29] and double sticky ovitraps (SOs) [30]. We retrospectively examined routine *Ae. aegypti* surveillance data collected by QH staff, consisting of BGS traps at 13 fixed locations [31] and SOs set at ca. 70 locales. QH also deployed BGS traps opportunistically to assess vector numbers at dengue case residences, although unfortunately they did not have sufficient resources to conduct expanded surveillance during the outbreak. Weather data were obtained from the Australian Bureau of Meteorology for Cairns Airport station located ca. 1 km. northwest of where the dengue outbreak started.

### Ethics

The Queensland Health Forensic and Scientific Services Human Ethics Committee reviewed the manuscript, with reference to Australia’s National Health and Medical Research Council’s National Statement on Ethical Conduct in Human Research. No ethical issues were identified with regard to collection of sera and use of data, and ethical approval to publish this paper has been granted. All virological samples were taken from the preexisting Queensland Health Forensic and Scientific Service’s collection and were anonymized. All dengue case data were obtained from QH dengue database held at Cairns and were anonymized. Informed consent to collect mosquitoes from properties was obtained from residents by Queensland Health staff.

### Viral Genome Sequencing

#### Viruses

DENV-3 was isolated from patients who had contracted locally acquired infections during each of the respective Cairns 1998 (accession number GenBank JN406514) and 2008 outbreaks (accession number 2008a - GenBank JN406515). The 1998 DENV-3 was used as a comparator of an earlier DENV-3 strain that resulted in a severe outbreak in the Cairns region, and is not meant to be a control in terms of incubation periods). The two DENV-3 isolates selected for phenotyping, designated Cairns 1998 and Cairns 2008a, were grown in C6/36 *Aedes albopictus* cells in Opti-MEM® reduced serum growth medium (GM; Gibco BRL®, Invitrogen, California) supplemented with 0.2% bovine serum albumin (Gibco BRL®, Invitrogen, California) at 28°C. To produce the virus stocks for the vector competence experiments, passage 4 viruses were harvested and pelleted by high speed centrifugation for 17 hours at 10,000 x *g* before resuspension in Opti-MEM® reduced serum medium supplemented with 10% foetal bovine serum (FBS; Gibco BRL®, Invitrogen, Australia).

#### RNA extraction and nucleotide sequencing

Viral RNA was extracted from 140 µL of infected C6/36 culture supernatant using the QIAmp Viral RNA Extraction Kit (Qiagen, Germany) according to the manufacturer’s instructions. Full-length genome amplification and sequencing was performed using the Superscript® III One-Step reverse transcription, polymerase chain reaction (RT-PCR) System with Platinum® *Taq* High Fidelity (Invitrogen Life Technologies, California) according to the manufacturer’s instructions, specific DENV-3 PCR primers (Pyke A.T., unpublished data) and the Big Dye® Terminator v3.1 cycle sequencing kit (Applied Biosystems, U.S.A) using the supplier’s protocols. Sequencing of the respective 5′ and 3′ untranslated regions was performed using the 5′/3′ RACE Kit (Roche Applied Science, U.S.A) and methods provided by the manufacturer.

#### Sequence and phylogenetic analyses

Nucleotide sequences were aligned with publicly available samples using TranslatorX [32] and confirmed by hand-inspection using Se-Al v2.0a11 software [Rambaut 1996; http://evolve.zoo.ox.ac.uk/. Accessed 10 Sept 2011]. Maximum-likelihood (ML)-based phylogenetic analysis was implemented using RAxML Black Box webserver [33] under the GTR+I+ Γ_ 4_ model of evolution as selected by jModeltest 0.1.1 [34]. Phylogenetic support was simultaneously generated in RAxML based on 100 ML replicates under the same model of evolution.

### Vector Competence Experiments

Characterization of the Cairns 1998 and Cairns 2008 DENV-3 in *Ae. aegypti* involved examining: a) susceptibility to infection; b) infection, dissemination and transmission rates; and c) the length of the extrinsic incubation period (EIP) to determine the day on which transmission first occurs. Five to seven day old F_2_ female *Ae. aegypti* collected from Cairns were exposed to blood meals containing DENV-3 stock, using a membrane feeding apparatus [35]. For the susceptibility trials, serial dilutions of the stock virus in the blood meal were prepared. For the other experiments, mosquitoes were exposed to a single dose of virus only. Blood engorged mosquitoes were maintained on 10% sucrose at 28°C, 75% RH and 12∶12 L:D (light:dark).

After 14 days, the modified capillary tube method of Aitken [36] was used to demonstrate transmission of the virus in remaining mosquitoes. To determine the length of the EIP, transmission was attempted using the capillary tube technique, and a sugar-soaked substrate technique [37]. On days 0–6, 9 and 13, individual mosquitoes were placed separately in 50 mL vials. A 1 cm square of honey-soaked filter paper card (FP; Bio-Rad, Hercules, CA) was placed over a hole that had been cut in the lid of the vial. After approx. 24 hr, each mosquito was then induced to expectorate into a capillary tube as described above. All whole bodies, body remnants, legs and wings, saliva expectorates, and honey-soaked filter cards were stored at −80°C.

#### Virus assay

The infectious blood meals were titrated as 10-fold dilutions in the wells of a 96 well microtiter plate seeded with confluent monolayers of C6/36 cells. Plates were incubated at 28°C for 10 days before being fixed with PBS/acetone. The mosquito whole bodies were homogenized separately from legs and wings in 1 mL of GM +3% FBS containing antibiotics and antimycotics using sterile glass beads. The homogenates and the saliva samples were filtered through a 0.2 µm Supor® membrane filter (Pall Corporation, Ann Arbor, MI). Filtrates were inoculated in duplicate into the wells of a 96 well microtiter plate seeded with confluent monolayers of C6/36 cells, which were then incubated and fixed as described above.

Virus infection was identified in the fixed cell monolayers using a cell culture enzyme immunoassay (CCEI) [38]. The flavivirus-reactive monoclonal antibody, 4G2 (TropBio, Townsville, Australia), was used as the primary antibody.

Viral RNA expectorated on the honey-soaked filter cards was detected using a real-time TaqMan RT-PCR assay [39] with the following modifications to the primers and probe: forward primer (D3UTRfor) 5′-AAGGACTAGAGGTTAGAGGAGACCC-3′, reverse primer (D3UTRrev) 5′- CGTTCTGTGCCTGGAATGATG -3′ and fluorogenic probe (D3UTR) 5′ FAM- AACAGCATATTGACGCTGGGAGAGACCAGA-TAMRA 3′. The RNA was eluted from the cards and extracted as described previously [37]. The Taqman RT-PCR was performed using the ABI 7500 Fast Real-Time PCR System (PE Applied Biosystems, U.S.A.). Detection of DENV-3 RNA and amplification of the 107 bp product was carried out using a single-tube, one-step RT-PCR format in a final reaction volume of 20 µL. The reaction mix was prepared using the Superscript III Platinum one-step qRT-PCR system (Invitrogen, U.S.A.) and contained 0.4 µL Superscript™ III RT/Platinum® *Taq* mix, 9.5 µL of 2X reaction mix, 300 nM primers, 150 nM dual-labelled probe, 47 nM ROX Reference Dye and 5 µL of extracted viral RNA or diluted synthetic control. The cycling conditions were as recommended by the manufacturer (Fast Mode) and consisted of one cycle at 50°C for 5 min, one cycle at 95°C for 10 min and 40 cycles at 95°C for 3 sec and 60°C for 30 sec. The threshold cycle number (*C*_t_) was determined for each sample and a negative result indicating no RNA detection corresponded to any *C*_t_ value which was ≥40 cycles.

#### Analysis

The susceptibility of *Ae. aegypti* to infection with the two DENV-3 strains was calculated by Probit analysis, and was expressed as ID_50_ and defined as the virus dose per mL at which 50% of mosquitoes tested positive for DENV-3 infection in the CCEIA (SPSS. SPSS for Windows, Rel. 16.0.0. Chicago: SPSS Inc.; 2007).

Day 14 infection, dissemination and transmission rates for *Ae. aegypti* exposed to the two DENV-3 strains were compared using the Fisher exact test.

In addition, effective vector competence (EVC) rates were calculated as in [40]. Briefly, EVC is calculated by determining the rate of change of vector competence over time in combination with the survival function from the traditional vectorial capacity equation to weight vector competence in terms of vector mortality. Here the daily survival rate was held constant at 0.90; the *Ae aegypti* model CIMSiM uses 0.91 as the nominal value for adult female survival [41]. From this, an EVC curve was produced and the area under this curve gives a measure of the cumulative vector competence for a period of time weighted by mortality. Using MCMC methods to calculate confidence intervals, we then determined whether areas under the EVC curves were statistically different [40].

## Results

### Environmental Conditions

**Figure 1:**
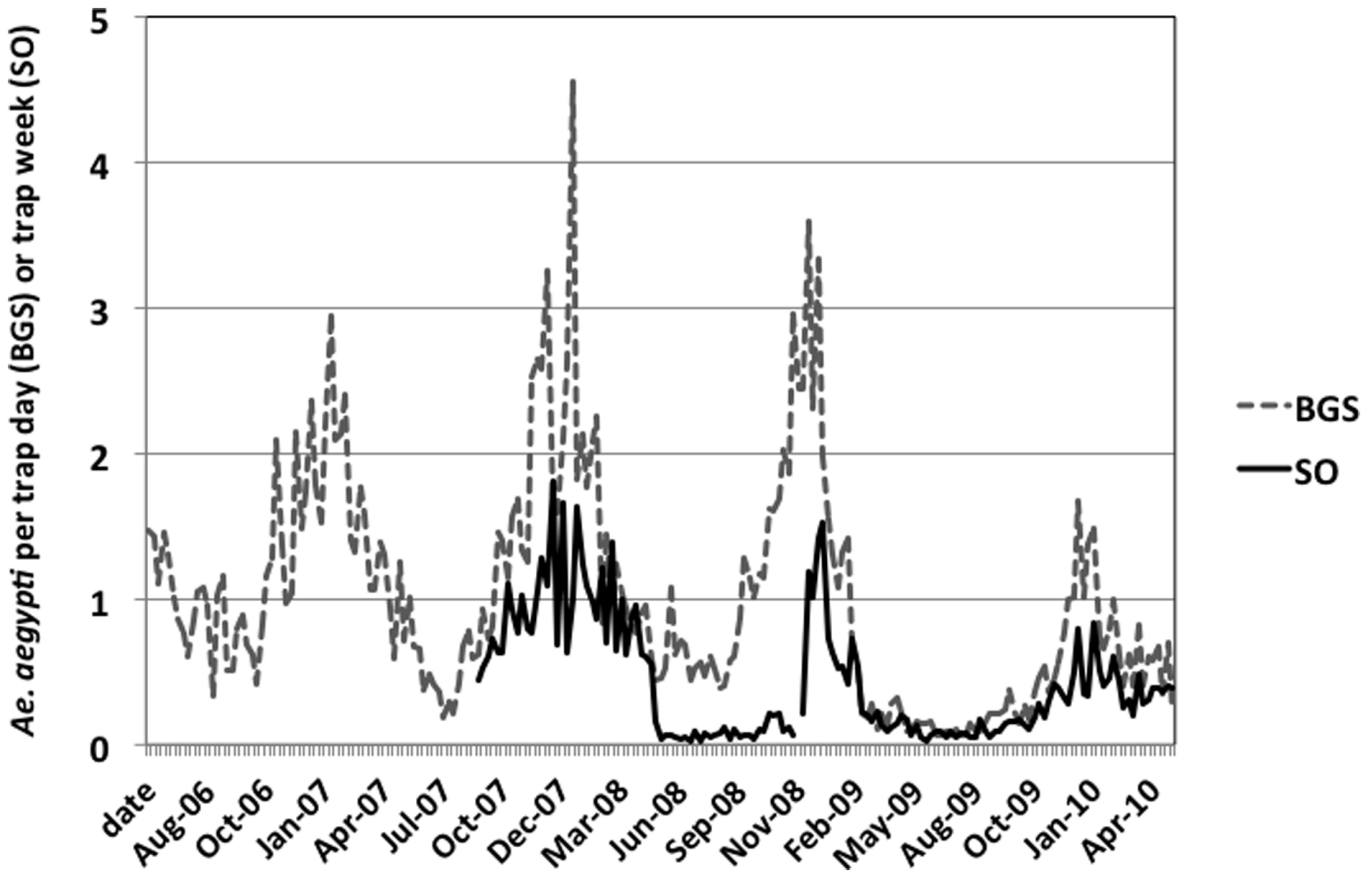
*Aedes aegypti* populations during the epidemic. Collection of female *Aedes aegypti* in sticky ovitraps (*n* = 64) and Biogents sentinel traps (BGS, *n* = 14) set in Cairns, Australia. Low SO collections from May-Nov. 2008 reflect poor capture by a dry glue temporally used in the trap.

Weather conditions prior to and during the epidemic were favorable for the production of *Ae. aegypti.* The Cairns dry season (May – November) generally has low rainfall, with an average of 133 mm of rain for the period from July to October. However, in 2008, rainfall during this period totaled 172 mm, with rain events >25 mm in July, September and October 2008. Heavy rains in late Sept. - mid Oct. (121 mm from 4 events) could have hatched *Ae. aegypti* eggs leading to the rapid escalation in *Ae. aegypti* numbers collected in BGSs traps and SOs in November (Fig. 1). Overall, mean collections of *Ae. aegypti* in both trapping methods were not unusually high for the early wet season, with mean female *Ae aegypti* collections reaching typical wet season peaks of 2 per BGS trap by Dec. 2008 ([31] and Fig. 1). However, foci of high populations existed. For instance, from 7 BGS traps set in Cairns North, the suburb where the epidemic started, a mean of 12.7 *Ae. aegypti* per trap day were collected in Dec. 2008, with 58 collected from a single trap.

Unusually warm weather occurred during November 2008, the first month of the outbreak, which could have enhanced dengue transmission. During Nov. 2008, the mean daily temperature was 28.0°C, which is 1.5°C above the 1941–2011 mean daily temperature for November in Cairns. Importantly, during a three day period from 22–24 November the daily high temperature and daily mean was 35.8°C and 30.8°C, respectively. Daily mean temperatures fell to normal levels in February-March, averaging about 27.5°C. Monthly rainfall from Nov. 2008– March 2009 was 58, 165, 868, 620 and 155 mm, respectively. This consistent rainfall should have provided sufficient precipitation for persistent production of *Ae. aegypti* in artificial containers.

### Epidemiological Factors

**Figure 2:**
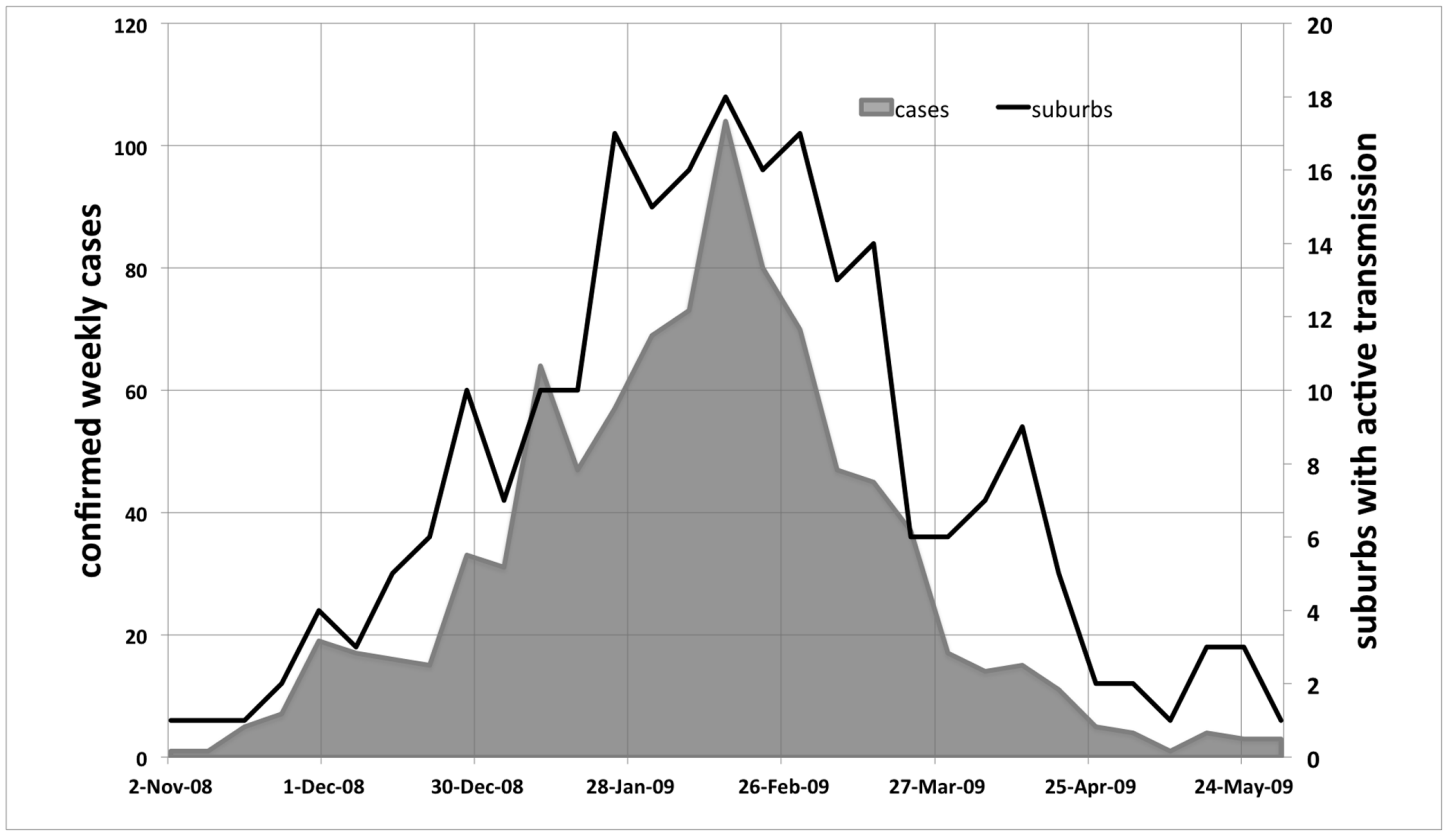
Epidemic curve of the DENV-3 outbreak. Number of confirmed cases of DENV-3 in Cairns region by week, and number of suburbs with active weekly transmission.

Health authorities were not notified of dengue activity in Cairns North until 28 Nov. 2008, with vector control initiated on 1 Dec. Active case finding retrospectively identified a resident (our purported index case), residing within the initial cluster of cases, who had visited Kalimantan, Indonesia in October 2008. This person returned to Cairns by 3 Nov. and became symptomatic on 5 Nov. 2008. It was strongly suspected that this individual imported the virus and initiated the outbreak as there had been no reported cases of DENV-3 in Cairns since the 1998 outbreak. A subsequent positive IgM ELISA test to dengue confirmed the case. The first pulse of locally-acquired DENV-3 cases (10–26 Nov.) were reported within 200 m of the as yet unknown suspected index case’s residence. Alarmingly, a total of 27 days had elapsed since the index case was viremic in Australia (4 November) and vector control was initiated (1 December). Dengue activity then rapidly spread throughout much of the Cairns region (Fig. 2).

Analysis of patient histories, and in particular, details of travel, suggested that the virus was transmitted from human to human exceptionally fast. The extrinsic incubation period (EIP) of dengue viruses is reported to range from 10–14 days [42] but varies with temperature and virus titers in viremic humans [13]. Potential EIPs, based upon modeling of vector competence experiments, suggests that the EIP of DENV could be as low as 5 and 2 days at 25°C and 30°C, respectively [43]. The intrinsic incubation period (IIP) in humans ranges from 3–14 days (average 4.5–7 days) [44]. Summing the EIP and the IIP creates a total incubation period (TIP) of 13–21 days. This value can be approximated by the period between successive rounds of transmission. For example, the time from onset of symptoms of the imported case and the first locally acquired case was 16 days for an outbreak of DENV-1 in Cairns in 2012 (G. Devine, personal communication), 17 days for DENV-2 in 2003 [9] and 18 days for a cluster of cases on Lizard Island in 1998 for the DENV-3 virus used in our vector competence studies. However, in the initial stages of the 2008–09 DENV-3 outbreak, there were already 6 locally acquired cases by 17 days (1 case each on day 10, 14, 15, and 16; 2 on day 17). In three instances we were able to identify the first new cases in what were previously uninfected suburbs, each of which were a considerable distance from suburbs with active transmission. Upon interview by a public health nurse, neither the suspected case, which initiated a new focus of transmission, nor the first recognized new case, reported that other householders or neighbors were ill. Each of these patients had a different scenario which contributed to the outbreak: a) the first locally-acquired case, who was a neighbor of the index case; b) an autoworker who was infected near the imported case, and then infected 4 co-workers at his workplace 2 km away; and c) a laborer who contracted dengue in Cairns and then infected a neighbour in Aloomba, an isolated hamlet (pop. 317) 15 km south of Cairns. Retrospective analysis of the time from onset of the locally imported case until onset of the first locally acquired case was 10, 9 and 11 days, respectively. We realize that this could be confounded by cryptic transmission, but the circumstances are supportive. Importantly, in each case there was no evidence of dengue transmission in the previous year within 2 km of where the imported case introduced dengue.

These observations suggest that the total incubation period of the DENV-3 affecting the Cairns region was relatively short. Considering a low IIP of 3–4 days, an EIP of ca. 6–7 days during a period of average daily temperatures of 28–29°C could have led to the rapid amplification and spread of the virus, with no obvious gap between the imported case and subsequent rounds of weekly dengue transmission (Fig. 2). Transmission was especially intense during the first month of the epidemic; with the biweekly effective reproduction number (R*t*; the average number of secondary cases per primary case at time *t*) estimated at 12 and 3.1 [5]. This is higher than R*t* values of 4.5 and 7.1 for the DENV-2 epidemic in Cairns in 2004 [5], which did not overwhelm control resources to the same degree [9].

The epidemic curve also shows that DENV-3 activity spread rapidly throughout the Cairns region. Indeed, by 1 January 2009, 2 months after the initiation of the outbreak, and 1 month after vector control commenced, dengue was active in at least 10 different suburbs (Fig. 2). The number of weekly confirmed cases rapidly increased during the holiday period in late December-January, and again in February, before the rate of infection dropped precipitously in March and April. Initial vector control activities included treatment of water-holding containers with methoprene, and IRS inside premises, supplemented with the deployment of lethal ovitraps to control adult *Ae. aegypti* [44], [45]. Detailed analysis of the impact of mosquito control on dengue transmission during the 2008–09 epidemic will be presented in another paper.

### The Collapse of the Epidemic

After declaration of the epidemic in January 2009, QH employed additional vector control personnel. When control activities were at their peak in February – April 2009, up to 50 vector control officers were actively engaged in in the field. QH also implemented a disaster response plan that involved the formulation of an incident management team. After peaking at a high of 104 cases per week in mid February 2009, transmission of dengue declined rapidly (Fig. 2). The onset of the last case was 31 May 2009 and the epidemic was declared over 3 months later. A total of 915 cases of DENV-3 were confirmed in the Cairns region, 6 cases fulfilled WHO criteria for dengue hemorrhagic fever (John McBride, personnel communication), and there was a single death on 4 March 2009.

#### Viral genome sequencing

In order to ascertain and compare molecular characteristics of the Cairns DENV-3 2008 strain, full-length genomic nucleotide sequencing was performed. Scrutiny of the nucleotide data revealed that the Cairns 2008a strain (**GenBank Accession number JN406515**) was most closely related to the previous 2004 Indonesian strain BA51 (**GenBank Accession number AY858037**) with 98.9% homology and only shared 93.1% homology with the Thailand-derived Cairns 1998 strain (**GenBank Accession number JN406514**). Similarly, at the amino acid level, the Cairns 2008 strain was 99.7% and 98.1% homologous to the Indonesian BA51 2004 and Cairns 1998 strains respectively. The Cairns 2008a virus was identical to a 2008 Indonesia isolate (strain 94, GenBank Accession number ) for the envelope (E) gene (1479 bp).

#### Phylogenetic Analysis

**Figure 3:**
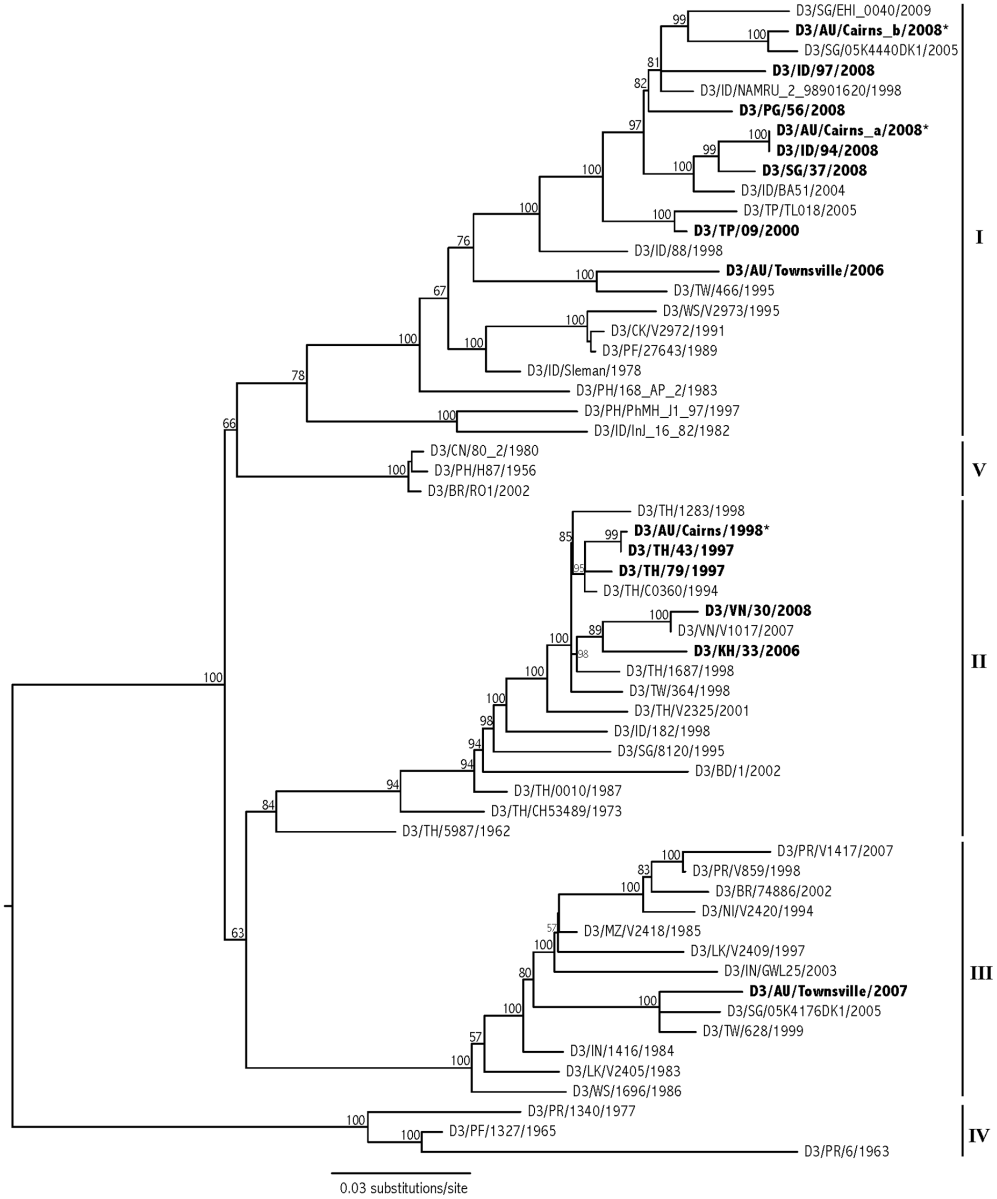
Phylogeny of DENV-3 from Cairns, Australia. Maximum likelihood (ML) phylogeny of DENV-3 from Cairns, Australia, from 1998 and 2008 (in bold, with asterix), based on whole-genome sequences. Several other isolates from the region were also sequenced and included (shown in bold, GenBank Accession numbers JN575563-80), along with publicly available sequences (accession numbers available upon request). Analysis was implemented in RAxML Black Box webserver [33] under the GTR+I+G model of evolution, including support at nodes based on 100 ML replicates. Sample labeling includes serotype/ISO country code/strain/year of collection. Genotypes I through V are indicated on the far right.

To establish the phylogenetic relationship between the Cairns DENV-3 2008 strain and other DENV-3 viruses and determine its likely geographical origin, a phylogenetic tree was constructed. Whole genome sequences of Cairns 2008 viruses, from isolates obtained from human sera during the 1^st^ month of the outbreak (Nov. 2008), were compared with 12 other DENV-3 isolates from locally imported cases, including the 1998 Cairns virus, along with sequences retrieved from GenBank (Genotypes I to V). The Cairns 2008 viruses grouped with other Genotype I DENV-3 viruses and were very distinct from the Cairns 1998 strain, which was shown to belong to Genotype II (Fig. 3). Genotype II was not observed in our study after 1998, and appears to be restricted within southeast Asia. More recent dengue activity in Queensland, Australia, based on our sequence data from both Cairns and Townsville between 2006 and 2008, involves the circulation of genotypes I and III (Fig. 3). Genotype I viruses are widespread throughout the region, including nearby East Timor, Papua New Guinea, and the Philippines, and accounted for the 2008 outbreak in Cairns. Strain Cairns 2008a, used in the vector competence experiments, was most closely related to a 2008 Indonesian isolate (100% ML bootstrap support, Fig. 3), supporting the epidemiologic link made from case interviews.

The two strains under comparison for different transmission kinetics differ by many genetic substitutions: 7% at the nucleotide level and 2% at the amino acid level, distributed throughout the genome. The amino acid differences are particular candidates for conferring phenotypic changes, any or all of which could affect the transmission potential. One amino acid difference at site 2062, which occurs in the NS3 gene and involved a change from arginine (Cairns 1998) to lysine (Cairns_a 2008), was under positive selection across the alignment according to selection analyses. Selection analyses were implemented on the Adaptive Evolution server at UC San Diego (http://www.datamonkey.org/).

### Vector Competence Experiments

#### Susceptibility to infection

The susceptibility of *Ae. aegypti* to infection with the 1997–98 and 2008–09 DENV-3 strains, expressed as Probit ID_50_ (SPSS 2007), was 10^5.3^(10^5.0^–10^5.6^, 95% CL) CCID_50_ per mL (χ^2^ = 2.98, df = 2, *P*>0.05) and 10^5.7^(10^5.4^–10^6.0^, 95% CL) CCID_50_ per mL (χ^2^ = 1.88, df = 2, *P*>0.05), respectively.

#### Infection, dissemination and transmission

When exposed to titers ranging from 10^5.2^ to 10^7.8^ CCID_50_ per mL, infection and dissemination rates were higher, and in some cases significantly higher, in *Ae. aegypti* exposed to the 2008/09 strain than those exposed to the 1997/98 strain (Table 1). However, at titers ≥10^6.3^, transmission rates were higher for the 1997/98 strain, and significantly higher in one trial, when compared to the 2008/09 strain.

**Table 1:** Infection, dissemination and transmission rates in Cairns *Ae. aegypti* 14 days after exposure to 1997/98 and 2008/09 strains of dengue virus type 3, as determined using a cell culture-enzyme immunoassay and the monoclonal antibody, 4G2.

Experiment	Virus	Virus titer^a^	% infection^b^		% dissemination^c^		% transmission^d^	
A	1997/98	5.2	15	(3/20)	15	(3/20)	0	(0/20)
	2008/09	6.0	70	(14/20)	65	(13/20)	10	(2/20)
		*P* value^e^		*P* = 0.001		*P* = 0.003		*P* = 0.487
B	1997/98	6.3	92	(23/25)	92	(23/25)	48	(12/25)
	2008/09	7.2	100	(25/25)	96	(24/25)	16	(4/25)
		*P* value^e^		*P* = 0.489		*P* = 1.000		*P* = 0.032
C	1997/98	7.6	84	(21/25)	80	(20/25)	32	(8/25)
	2008/09	7.8	100	(25/25)	100	(25/25)	20	(5/25)
		*P* value^e^		*P* = 0.109		*P* = 0.050		*P* = 0.520

See Materials and Methods section for details on conduct of the three trials.

a Titer (log_10_CCID_50_/mL) of the infectious blood meal to which mosquitoes were exposed.

b Percentage of mosquitoes containing virus in their bodies (number positive/number tested).

c Percentage of mosquitoes containing virus in their legs and wings (number positive/number tested).

d Percentage of mosquito expectorates in which virus was detected (number of positive expectorates/number tested).

e *P* value calculated by Fisher’s Exact test.

#### Length of the extrinsic incubation period

For the time series experiment, disseminated virus was first detected on day two for the 1997/98 strain and on day 4 for the 2008/09 strain (Table 2). Transmission occurred first in the mosquitoes exposed to the 2008/09 strain, when on day 5 post exposure, 2/20 and 3/20 mosquitoes transmitted the virus via the capillary tube and honey-baited FP, respectively. Transmission of the 1997/98 strain was first observed on day six when 3/20 mosquitoes expectorated virus on the honey-baited FP. Transmission peaked at 25% for the 2008/09 strain on day seven and did not increase after this day. In contrast, the highest transmission rates observed with the 1997/98 strain was 32% and 40%, with the capillary tube and honey-baited FP, respectively, at day 14 post exposure.

**Table 2:** Infection, dissemination and transmission rates in Cairns *Ae. aegypti* on various days post exposure (PE) to the 1997/98 and 2008/09 strains of dengue virus type 3.

	2008/09 DENV-3 (10^5.1^ CCID_50_/mosquito)								1997/98 DENV-3 (10^4.9^ CCID_50_/mosquito)							
Day PI	% infection^a^		% dissem.^b^		% trans.^c^		% trans.^d^		% infection^a^		% dissem.^b^		% trans.^c^		% trans.^d^	
0	100	(20/20)	0	(0/20)	0	(0/20)	0	(0/20)	95	(19/20)	0	(0/20)	0	(0/20)	0	(0/20)
1	80	(16/20)	0	(0/20)	0	(0/20)	0	(0/20)	100	(20/20)	0	(0/20)	0	(0/20)	0	(0/20)
2	95	(19/20)	0	(0/20)	0	(0/20)	0	(0/20)	100	(20/20)	5	(1/20)	0	(0/20)	0	(0/20)
3	55	(11/20)	0	(0/20)	0	(0/20)	0	(0/20)	95	(19/20)	10	(2/20)	0	(0/20)	0	(0/20)
4	90	(18/20)	45	(9/20)	0	(0/20)	0	(0/20)	85	(17/20)	0	(0/20)	0	(0/20)	0	(0/20)
5	95	(19/20)	25	(5/20)	10	(2/20)	15	(3/20)	100	(20/20)	60	(12/20)	0	(0/20)	0	(0/20)
6	95	(19/20)	80	(16/20)	5	(1/20)	10	(2/20)	90	(18/20)	65	(13/20)	0	(0/20)	15	(3/20)
7	100	(20/20)	85	(17/20)	0	(0/20)	25	(5/20)	85	(17/20)	70	(14/20)	10	(2/20)	15	(3/20)
10	100	(20/20)	100	(20/20)	10	(2/20)	24	(4/17)	90	(18/20)	84	(16/19)	11	(2/19)	20	(4/20)
14	100	(25/25)	100	(25/25)	20	(5/25)	24	(6/25)	84	(21/25)	80	(20/25)	32	(8/25)	40	(10/25)

Shaded rows denote the first day post infection day that transmission was observed.

a Percentage of mosquitoes containing virus in their bodies (number positive/number tested); days 0–1 likely represent infected blood bolus.

b Percentage of mosquitoes containing virus in their legs and wings (number positive/number tested).

a Percentage of saliva expectorates collected using the capillary tube method of [**UNRESOLVED**] that were positive by cell culture-enzyme immunoassay (number positive/number tested).

b Percentage of saliva expectorates collected using the honey-baited filter paper method of Hall-Mendelin et al. [37] that were positive by TaqMan RT-PCR (number positive/number tested).

**Figure 4:**
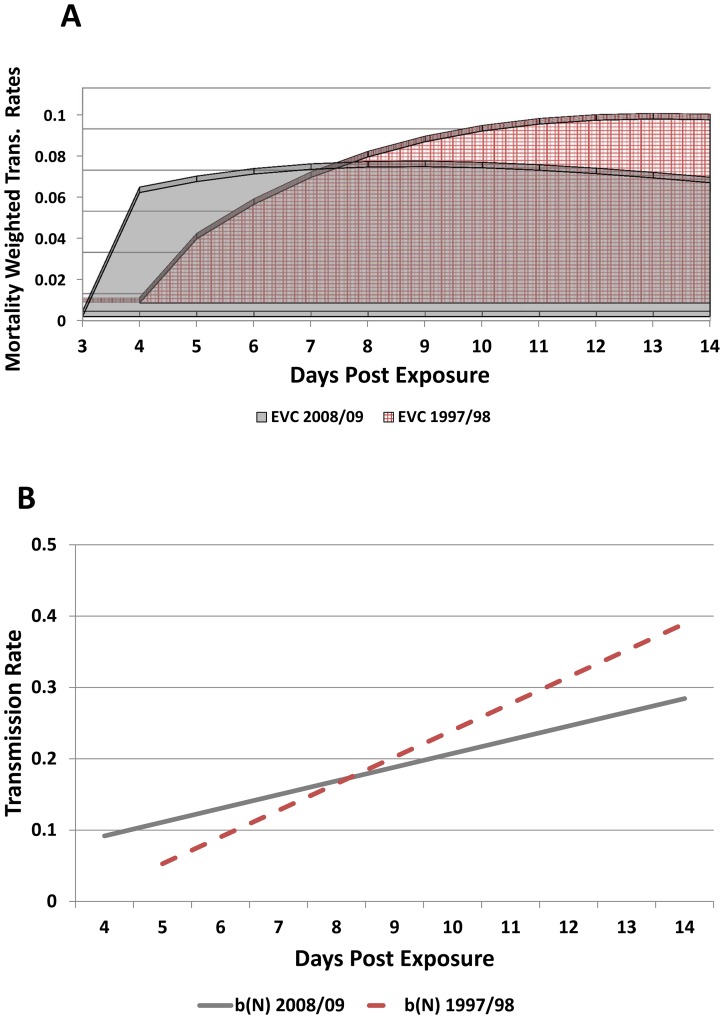
Effective vector competence of the Cairns DENV-3. Top: The change in vector competence over time through a population of vectors is a dynamic process. Here the difference in dynamic vector competence between the 2008/09 (dashed line) and 1997/98 (solid line) strains is shown. These linear functions then replace the static measure of vector competence and are used to calculate the effective vector competence (EVC). Bottom: EVC curves for the two strains- 2008/09 (foreground, solid) and 1997/98 (background, checkered)- are shown. These curves utilize the dynamic vector competence function over time and also weight each day’s value for the effect of mosquito mortality.

The effective vector competence (EVC) values for the 2008/09 strain was 69.61%; and for the 1997/98 strain (Fig. 4), EVC was calculated as 66.92%, showing that the 2008/09 strain did have a slight advantage because of its shorter EIP, though this difference was not statistically significant. Given the disparity in the ultimate transmission rates of these two strains, where the 1997/98 strain reached rates 1.6 times that of the 2008/09 strain, the fact that EVC values are so close demonstrates the potential importance of shorter EIP in transmission potential. That is, given the much higher transmission rates of the 1997/98 strain, if the shorter EIP of the 2008/09 strain was not important, we would expect the difference in the EVC values to be greater.

## Discussion

A combination of conditions in Nov. and Dec. 2008 may have enhanced dengue transmission in the Cairns North suburb where the outbreak started. Rainfall in the winter and again in late Sept. – Oct. would have flooded containers and facilitated *Ae. aegypti* production which peaked during December. The period of high temperatures during Nov. 2008 would have probably reduced the EIP in mosquitoes [13], [14], resulting in a potential escalation of dengue transmission. Hot weather continued through December 2008 when the mean daily temperature was 28.6°C, which is 1°C above average. Heat waves have previously been associated with epidemic transmission of dengue in Sumatra [47], Indonesia [48], the Caribbean [49], and the South Pacific [50]. This period of high temperature would have had a multifaceted impact on mosquito physiology, whereby higher temperatures increase *Ae. aegypti* biting rates [51] and reduce the length of the gonotrophic cycle [14], potentially leading to a higher frequency of contact between infected mosquitoes and humans.

Perhaps the most crucial factor that led to the explosiveness of the epidemic was the apparently short EIP of the 2008–09 DENV-3 strain, whereby transmission was demonstrated only 5 days after mosquitoes fed on a viraemic blood meal. The EIP of DENV is temperature dependent, with higher temperature increasing the rate of virus dissemination and subsequent transmission [14]. Therefore, it is possible that higher temperatures in late November 2008 (ca. 30°C) may have resulted in an even shorter EIP than the 5 days we observed at laboratory temperature of 28°C. The EIP temperature was maintained at 28°C in our experiments to allow for a direct comparison between virus strains without the compounding influence of temperature. Although a previous study observed infected salivary glands after 4 days [52], we are aware of no reported instances of DENVs being transmitted this rapidly, irrespective of the temperature that mosquitoes were maintained at. While the EIP of dengue viruses is typically reported to range from 8–12 days, a recent study suggests that there is more variability, and the extreme low range is 2–5 days [43].

Importantly, when daily transmission rates were included in the EVC model, it was revealed that transmission of the 2008/09 strain a day earlier had the potential to cause a larger outbreak than the 1997/98 strain. This is despite Cairns *Ae. aegypti* being more susceptible to the latter strain and ultimately transmitting it at a higher rate than the 2008/09 strain. Thus, we conclude that the 1 day shorter EIP rescued the population-level transmission potential of the 2008/09 strain even though the transmission rate is 1.6 times less than the 1997/98 strain. This phenotype of rapid virus transmission is supported by epidemiological data. We identified 3 likely isolated transmission scenarios where the TIP was 9, 10 and 11 days. Furthermore, Rt for the first 4 weeks of transmission was 12 and 3.1, higher than transmission rates for the 2003 DENV-2 epidemic.

A reduced EIP has important epidemiological implications. A reduced EIP with CHIKV in *Aedes albopictus* of 4–5 days resulted in a massive epidemic of Reunion Island [18]. Similarly, the reduced EIP exhibited by the WN02 genotype has been a factor in the replacement of NY99 as the dominant of West Nile virus genotype in the USA [53]. Similar to these two viruses, the rapid transmission of DENV would also allow for a larger number of mosquitoes to transmit. With a nominal daily survival of 0.90 for female *Ae. aegypti* [41], a conservative reduction in the EIP from 8 days to 5 days would allow for up to 37% more mosquitoes to potentially transmit dengue. Of course, as the vector competence experiments demonstrate, not all individuals get infected, nor are able to transmit so quickly.

The EVC calculations provide evidence for the advantage of a shorter EIP. Traditional vector competence measurements are often static and taken at longer EIPs, discounting time to transmission in the overall calculation of transmission potential. A static transmission rate of 24% (Table 2, 14 days) would not suggest such an explosive transmission potential as seen with the 2008/09 strain in Cairns. The effective vector competence results suggest that the 2008/09 shorter EIP is enough to compensate for its lack of overall transmission rates, as we show by comparing it to a strain with much higher transmission rates (1.6 times higher at the highest titer). This suggests not only a means to explain how the 2008/09 strain spread so quickly, but also suggests that multiple replicative strategies exist among dengue strains. For example, a virus strain may become transmissible more slowly (longer EIP) but attain an overall higher transmission rate due to higher transmission rates in the mosquito (exhibited by the 1997/98 strain). Alternatively, a viral strain may become transmissible more quickly (with a shorter EIP such as the 2008/09 strain) that can compensate for a lower overall rate of transmission in mosquitoes. Certainly, beyond these examples other fitness scenarios are likely, which could impact transmission intensity. These two strains of DENV-3 exhibited two distinct patterns of dissemination yet achieved near-identical effective vector competence values, suggesting two divergent fitness strategies and potentially accounting the rapid and intense transmission of the 2008/09 strain despite its lower ultimate transmission rate.

While the outbreak appears to have been initiated by the purported index case from Kalimantan, it is possible that multiple introductions of DENV-3 may have occurred. However, the multiple DENV-3s detected in Cairns in 2008–09, Cairns_b 2008 was distinct from Cairns a 2008 both in that the former was part of a distinct incident of local transmission in the nearby community of Port Douglas Queensland earlier in February of 2008, whereas the latter was sampled in late November, 2008, as part of the epidemic described in this study. The phylogenetic analysis further indicates that the Cairns 2008 samples are different, with closest relatives in the tree from Indonesia 2008 (Cairns a) and Singapore 2005 (Cairns b) representing separate incursion events. The association of Cairns b 2008 with an older Singapore isolate as opposed to a more recent one simply reflects the sparse sampling of dengue by sequencing in the region. Even if unknown introductions of DENV-3 did occur, this does not distract from the fact that high transmission during the initial 2 months of the outbreak directly contributed to the overall size of the epidemic, and that environmental conditions and virological characteristics of the virus resulted in the explosive early transmission.

**Figure 5:**
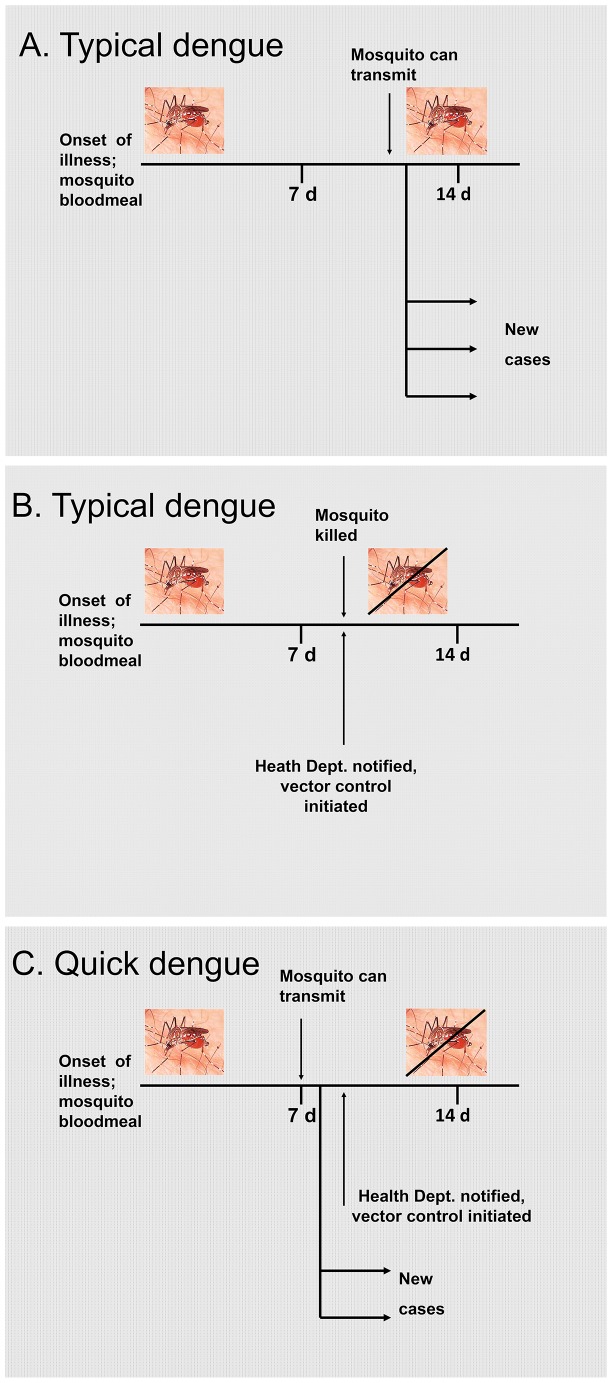
Proposed transmission cycle of the Cairns DENV-3. Schematic of transmission cycle (A) and intervention results (B) for typical dengue (EIP = 10 days) and quick dengue (C; EIP = 5 days).

Despite a relatively low transmission rate (from the vector competence experiments), rapid transmission of the virus could still have profound implications on its management, especially with high vector populations. A 2–4 day faster transmission cycle would create situations where there could already be a second round of transmission (i.e., mosquitoes feeding on a notified case would already be transmitting the virus) especially when the median delay in notification of a dengue case of 7 days is taken into consideration [53]. This would greatly compromise the ability to institute control measures, such as interior residual spraying to kill adult mosquitoes, in response to individual cases with a view to disrupting the transmission cycle (Fig. 5). Furthermore, delayed action control methods, such as lethal ovitraps (LOs) that kill gravid mosquitoes >5 days old [45], [46], would similarly be rendered ineffective by a rapidly transmitted strain of virus. Indeed, we had continued dengue transmission after deployment of LOs in Cairns North in Dec., and reverted to IRS for a more rapid insecticidal knockdown. While there was some evidence of tolerance of *Ae. aegypti* to bifenthrin used in the LOs, resistance testing did not detect physiological resistance to the insecticide (N. Endersby, University of Melbourne, unpublished data).

The delayed identification of dengue activity in November 2008 also contributed to the epidemic. Vector control was not initiated until 27 days after the index case became viremic. By this time, the third cycle of dengue transmission was occurring in Cairns North. Delays in case notification have been identified as a risk factor for dengue outbreaks in north Queensland [54], and helped trigger large outbreaks in 1997 [55] and 2003 [9]. Dengue control efforts were also hampered by the rapid geographic spread of the virus (Fig. 2). December and January are the Christmas holiday season in Cairns, and people often travel to friends and relatives. The human-mediated dispersal of dengue associated with the Christmas holiday season, the so-called ‘Christmas Rush’, is evident by the rapid escalation in both affected suburbs and active cases in late December-January (Fig. 2).

Low herd immunity would have also contributed to the explosive nature of the outbreak. While Cairns has regular outbreaks, most are small, with generally less than 100 confirmed cases. Analysis of blood donations from non-febrile residents during the epidemic indicated that IgG to DENVs was 10.1% (95% CI: 8.7–11.5%) [56], and as the Cairns region had not had an outbreak of DENV-3 since 1998, herd immunity to DENV-3 would be expected to be very low.

The epidemic collapsed rapidly following its peak in February (Fig. 2). Several key factors contributed to this rapid decline in cases. The epidemic received widespread media attention, so the public were well informed of their roles and responsibilities (removal of water-holding containers, and use of pyrethroid surface sprays indoors). A number of new strategies of control were also implemented. The Queensland State Emergency Service door knocked, and delivered information kits and cans of pyrethroid surface spray to residents in suburbs at risk. A SMS texting service sent messages to mobile phones warning residents of active DENV transmission within their residential area. An increased number of vector control teams conducted widespread source reduction and treatment of larger water-holding containers with *s*-methoprene pellets. Both BGS traps and sticky ovitraps (Fig. 1) depict a rapid decline in female *Ae. aegypti* populations in February – March 2009, reflecting the implementation of these enhanced control strategies. There was hardly any suburb in Cairns that had not reported cases of dengue, and had not been subject to vector control by April 2009. Thus, untreated suburbs with high mosquito populations were probably increasingly rare. The last active transmission of DENV-3 occurred in Earlville in May, where IRS teams rapidly responded and treated the residence and surrounding houses.

This outbreak highlights the impact that DENV strains with a reduced EIP can have on public health. The apparent speed of transmission and its rapid geographic spread overwhelmed what had been a successful, organized first world dengue control program. This outbreak also emphasises the need for a strategic shift in dengue control from a reactionary response to cases to a preventative approach to minimize vector populations. Since the outbreak, high-risk areas have been subject to preventative larval control and source reduction campaigns to suppress populations prior to the next wet season. Despite a record number of imported viremic dengue cases into north Queensland (51) and 4 distinct outbreaks in 2010, the total number of locally acquired cases was only 24. Commensurate with what has occurred previously with dengue, CHIKV and WNV, the 2008–09 Cairns DENV-3 epidemic suggests that small genetic changes in arboviruses might express altered phenotypes in humans and mosquitoes that potentially increase transmission creating outbreaks that can affect epidemic transmission and overwhelm public health programs [18]–[26]. It also emphasizes the important role that rapid case recognition and reporting, and effective preventative mosquito control programs, can play in minimizing the ability of these virus strains to escalate into a widespread epidemic. Finally, the occurrence of the closely related strains of DENV-3 serotype 1 in Cairns, Indonesia and Singapore from 2008 (Fig. 3) are suggestive of the evolution of new strains and rapid movement of dengue viruses regionally that initiate local outbreaks [57].
