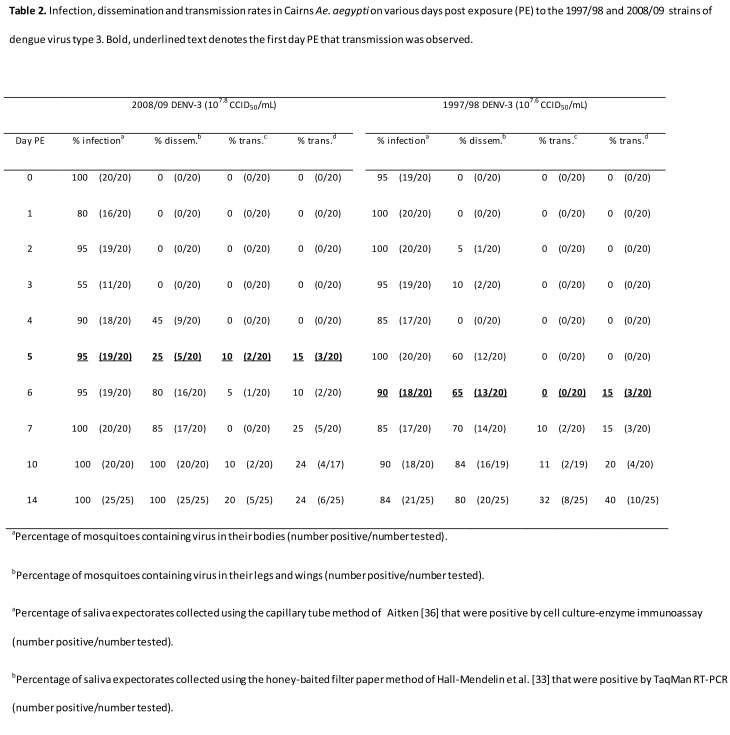# Correction: An Explosive Epidemic of DENV-3 in Cairns, Australia

**DOI:** 10.1371/annotation/a8dfd4ee-f4b7-443e-bf78-ebb0dab4e55b

**Published:** 2013-12-30

**Authors:** Scott A. Ritchie, Alyssa T. Pyke, Sonja Hall-Mendelin, Andrew Day, Christopher N. Mores, Rebecca C. Christofferson, Duane J. Gubler, Shannon N. Bennett, Andrew F. van den Hurk

There are several errors in Table 2. Please refer to the corrected version of Table 2 here: 

**Figure:**