# Correction: An Explosive Epidemic of DENV-3 in Cairns, Australia

**DOI:** 10.1371/journal.pone.0105846

**Published:** 2014-08-08

**Authors:** The PLOS ONE Staff

There is a missing grant number in the funding statement for this article. Please refer to the correct funding statement below:

Research at QHFSS was funded by an internal funds provided by Queensland Health and the US National Institutes of Health/NIGMS U01GM097661. The funders had no role in study design, data collection and analysis, decision to publish, or preparation of the manuscript.
